
JOURNAL INFORMATION
==============================
NLM Title Abbreviation: ScientificWorldJournal
Journal ID: ScientificWorldJournal
NLM Journal ID: 101131163
Journal ID: TSWJ

The Scientific World Journal

ISSN: 2356-6140
EISSN: 1537-744X
Publisher: Wiley

ARTICLE INFORMATION
==============================
PMCID: PMC5917234
PMID: 29861690
DOI: 10.1100/tsw.2006.392
Article ID: 561393
Article version: 1
Subjects: Editorial

Robotic Surgery in Urology: Special Issue

Steers William *
Hovey Dabney Professor and Chair, Department of Urology, University of Virginia School of Medicine, Charlottesville, VA 22908, USA
*William D. Steers: wds6t@virginia.eduAcademic Editor: William D. Steers

Publication date: 2006
Electronic publication date: 2006 Feb 17
Volume: 6
First page: 2529
Last page: 2530
Accepted 2006 Feb 6
Copyright: Copyright © 2006 William Steers.
Copyright year: 2006
License: This is an open access article distributed under the Creative Commons Attribution License, which permits unrestricted use, distribution, and reproduction in any medium, provided the original work is properly cited.
License URL: https://creativecommons.org/licenses/by/3.0/

==============================
Editorial
Robotic Surgery in Urology
*TheScientificWorldJOURNAL* (2006) 6, 2529–2530
TSW Urology
ISSN 1537-744X; DOI 10.1100/tsw.2006.392

TheScientificWorldJOURNAL
www.thescientificworld.com

### Robotic Surgery in Urology: Special Issue

William D. Steers

*Hovey Dabney Professor and Chair, Department of Urology, University of Virginia School of Medicine, Charlottesville, VA 22908; Tel: 434-924-9107*

**E-mail:** wds6t@virginia.edu

*Accepted February 6, 2006; Published February 17, 2006*

---

In 1976, while codirecting the Oliphant Lecturer series at Cornell University, our guest speaker, a biochemist and science fiction writer, discovered that I was a combined engineering and premed major with a keen interest in surgery. During a reception, he wandered over to me and in between some unrepeatable limericks, announced that one day surgery would be done by robots. At the time, I thought his prediction was far fetched and dismissed it; not as impossible, but as too short range. Now, only 30 years later, his uncanny prediction is a reality. My skepticism should have been tempered by the fact that the speaker was Issac Asimov, who coined the term “robotics” and although he may have envisioned a robot as a preprogrammed surgeon, his prediction was close enough to warrant my continued admiration.

This issue explores the rapidly escalating use of robotic-assisted laparoscopic surgery in urology. In the relatively short period of a couple of years, robotics has evolved from a curiosity to mainstream as judged by the volume of robotic procedures being performed. Urologists are early adopters of technology, so it is predictable that robotic surgery has taken off in our surgical specialty. Regardless of personal feelings as to its merits and costs, it is a platform for surgery that must be investigated. Criticism and scrutiny of objective outcomes may have softened for the wrong reasons as everyone jumps on the bandwagon to embrace robotics or lose patients. This issue of *TSW Urology* presents the current results and impressions regarding robotic surgery by world leaders. Unfortunately, missing are prospective randomized comparator trials. It would seem that as the boundaries of surgery expand, the reporting of outcomes fail to keep pace with changes in methodologies.

The papers included in this issue span topics ranging from the economics and logistics of setting up a program, akin to an open heart program in the 1970s, to technical details of robotic prostatectomy, cystectomy, pyeloplasty, and other pelvic surgeries. It is tempting to speculate where robotics is heading. Its future uses are intriguing and, if realized, will allow robotics to transform how we approach surgery in this 21$^{st}$ century. Currently, laparoscopic and robotic-assisted surgery merely duplicates procedures that can be done open. However, if one envisions combining *in vivo* cellular imaging, nanotechnology, and biologic markers with robotics, a leap forward into new procedures that truly outperform current surgeries becomes feasible. Robotics also conforms to its original definition of helping humans, but in ways not immediately obvious. Ergonomics for surgeons is a poorly studied area and may tip the balance in favor of robotics over pure laparoscopic approaches. Surgical simulation, poor for open surgery, may be more amenable using a robotic platform and virtual training. I like to use the hackneyed fighter pilot analogy for what promise robotics may hold for patients. In World War I, the best aces possessed the greatest dexterity, stamina, and enhanced vision. By analogy, some of our colleagues are aces. However, this surgical technology may permit a leveling of the playing field by minimizing the standard deviation for blood loss, side effects, and positive tumor margins. Indeed, one paper in this issue focuses on side effects. Although objective outcomes may be sparse and enthusiasm exaggerated, robotic techniques and instrumentation will improve. Continued research will lead to refinements that justify widespread

©2006 with author.
Published by TheScientificWorld; www.thescientificworld.com adoption and alter the standard of care. This progression from science fiction to reality should not tempt us to abandon a critical appraisal of exciting technology as we advance forward.

**This article should be cited as follows:**

Steers, W.D. (2006) Robotic surgery in urology: special issue. *TSW Urology* **1(S1),** 1–2. DOI 10.1100/tswurol.2006.49.
